# Bacterial Adhesion Strength on Titanium Surfaces Quantified by Atomic Force Microscopy: A Systematic Review

**DOI:** 10.3390/antibiotics12060994

**Published:** 2023-06-01

**Authors:** Juliana Dias Corpa Tardelli, Vanderlei Salvador Bagnato, Andréa Cândido dos Reis

**Affiliations:** 1 Department of Dental Materials and Prosthesis, School of Dentistry of Ribeirão Preto, University of São Paulo (USP), Ribeirão Preto 14040-904, Brazil; 2 Department of Physics and Materials Science, São Carlos Institute of Physics, University of São Paulo (USP), São Carlos 13566-970, Brazil

**Keywords:** titanium, dental implant, bacteria, atomic force microscopy, AFM, bacterial adhesion

## Abstract

Few studies have been able to elucidate the correlation of factors determining the strength of interaction between bacterial cells and substrate at the molecular level. The aim was to answer the following question: What biophysical factors should be considered when analyzing the bacterial adhesion strength on titanium surfaces and its alloys for implants quantified by atomic force microscopy? This review followed PRISMA. The search strategy was applied in four databases. The selection process was carried out in two stages. The risk of bias was analyzed. One thousand four hundred sixty-three articles were found. After removing the duplicates, 1126 were screened by title and abstract, of which 57 were selected for full reading and 5 were included; 3 had a low risk of bias and 2 moderated risks of bias. (1) The current literature shows the preference of bacteria to adhere to surfaces of the same hydrophilicity. However, this fact was contradicted by this systematic review, which demonstrated that hydrophobic bacteria developed hydrogen bonds and adhered to hydrophilic surfaces; (2) the application of surface treatments that induce the reduction of areas favorable for bacterial adhesion interfere more in the formation of biofilm than surface roughness; and (3) bacterial colonization should be evaluated in time-dependent studies as they develop adaptation mechanisms, related to time, which are obscure in this review.

## 1. Introduction

Peri-implant infection is the leading cause of the failure of implant-supported biomedical rehabilitation using titanium and its alloys [1,2,3,4,5,6,7,8,9,10]. Therefore, studies aim to understand the biophysical phenomena involved from the first moment of electrostatic interaction between the bacteria and the surface of the biomaterial to its adhesion and colonization mechanisms for the development of effective strategies that suppress the occurrence of infections [1,11,12,13]. In this way, understanding the biomolecular level is a promising antibiofilm regulation strategy, as it aims to unravel the bacterial adaptation mechanisms on the surface to prevent their development, unlike most existing surface treatments that focus on eradicating them [1,11,12,13].

Bacterial adhesion to the surface occurs in two stages, the first is permeated by nonspecific long-range forces, such as van der Waals and electrostatic, that dictate their attraction or repulsion [1,14,15,16,17,18,19,20]. The second is mediated by the specific forces of short-range bacterial receptors, pili, and capsules on the surface in an irreversible way, making bacterial detachment only possible with the interposition of mechanical or chemical action [1,14,15,16,17,18,19,20].

Biofilms are formed and matured on titanium surfaces due to the physicochemical conditions of the implant surface, chemical composition, topography, roughness, and wettability, which influence blood plasma protein adhesion, mediating bacterial adhesion [10,20,21,22]. After the adsorption of proteins on the substrate, primary bacterial colonization occurs through the recognition of protein receptors on the substrate, which select certain species, such as *Streptococcus oralis* and *Actinomyces naeslundii*, both Gram-positive, facultative anaerobes [21,23,24].

After primary colonization, the decrease in oxygen concentration favors the incorporation of secondary colonizers such as *Veillonella parvula* and *Fusobacterium nucleatum* [21,23,25]. This way, the coaggregation process occurs, where each bacterial strain aggregates with a specific bacterial set and promotes the coaggregation of late colonizers, such as *Porphyromonas gingivalis* obligate anaerobe of slow colonization [21,23,26]. The three-dimensional bacterial organization in a matrix consisting of polysaccharides, proteins, nucleic acids, and lipids determines the formation of the biofilm. It is noteworthy that after biofilm formation, bacterial resistance to the body’s defense mechanisms and antibiotic therapies is superior [15,17,20].

While studies demonstrate that the rough surface presents higher bacterial adhesion, as it provides a greater surface area [27,28,29,30], other studies report no correlation between roughness and bacterial retention capacity [31,32,33,34]. Ludecke et al., 2016 [35], demonstrated that the increase in roughness at a nanometric scale decreased the number of adhesion points and the consequent surface area, unlike the micro and macrometric scales, in which increased roughness provides a greater contact area.

The results present in the literature are also inconclusive and contradictory for wettability [30]. According to the review of Gittens et al., 2014 [36], bacteria prefer surfaces with the same hydrophilicity. Still, others report that surface hydrophobicity reduces bacterial velocity, promoting adhesion [30,37,38], while others report that water on the surface promotes hydrogen bonds with the bacteria because of its 70% water content [1,14,34].

The evaluation of bacterial adhesion strength on surfaces, in accord with the review by Alam et al., 2019 [39], can be evaluated by quantitative techniques, which measure the magnitude of bacterial adhesion force on the surface, as well as qualitatively when assessing the percentage of bacterial cells adhered to a surface. Among these methods, centrifugation, plate-and-wash technique, reflectance interference contrast microscopy, spinning disc assay, and buoyancy force technique are considered qualitative, and step pressure technique, nanoindentation, cyto detacher, optical tweezers, and atomic force microscopy (AFM) are considered quantitative [39].

According to the review by Alam et al., 2019 [39], atomic force microscopy is the most effective and suitable technique for studying cell–biomaterial interaction because it is able to measure the adhesion force of cells and biological molecules at the atomic level with high sensitivity (pN) on a three-dimensional nanometer scale [20,39,40,41,42]. The technique consists of a silicon or diamond nanometer tip that sweeps in constant contact, intermittent contact, or non-contact the sample surface in dimensions x, y, and z through a piezoelectric device [15,40,43,44,45].

The analysis of bacterial adhesion strength on the substrate is performed in two steps, the first is the preparation of the bacterial probe by immersing the tip in a solution of poly-l-lysine (PLL), polydopamine, polyethyleneimine, or glutaraldehyde so that the bacteria remain immobilized in the tip during the next step, which is the recording of the force–distance curve (F-D) [1,11,14,15,39]. The mechanism of this analysis consists of the following: (I) the approximation of the tip in the sample where the bacteria is, (II) the immobilization of the bacteria in the tip, and (III) the retraction of the tip of 0–10 s to record the F-D curvature that determines the force (10 pN to 1 μN) and adhesion energy [20,39,40,46,47]. The tip, after analysis, is stained with a fluorescent dye, and fluorescence microscopy is used to observe whether the bacteria is alive [39].

This systematic review aimed to evaluate the existing literature to answer the question “What biophysical factors should be considered when analyzing the bacterial adhesion strength on titanium surfaces and its alloys for implants quantified by atomic force microscopy?”

Because the bacterial adhesion force on titanium surfaces influences their survival on the substrate and directly interferes with the bacterial colonization process for the development of the biofilm; however, few studies aim to understand bacterial cell–substrate interaction at the molecular level.

## 2. Results

### 2.1. Result of the Selection Process

After the application of the personalized search strategy “(titanium OR “titanium alloy” OR “Ti alloy” OR “dental implant” OR implant) AND (“atomic force microscopy” OR “AFM” OR “atomic force spectroscopy” OR “simply force spectroscopy”) AND (“bacterial adhesion” OR “bacterial attachment”)” in the Embase, PubMed, Scopus, and Science Direct databases, 1263 articles were found.

**Figure 1:**
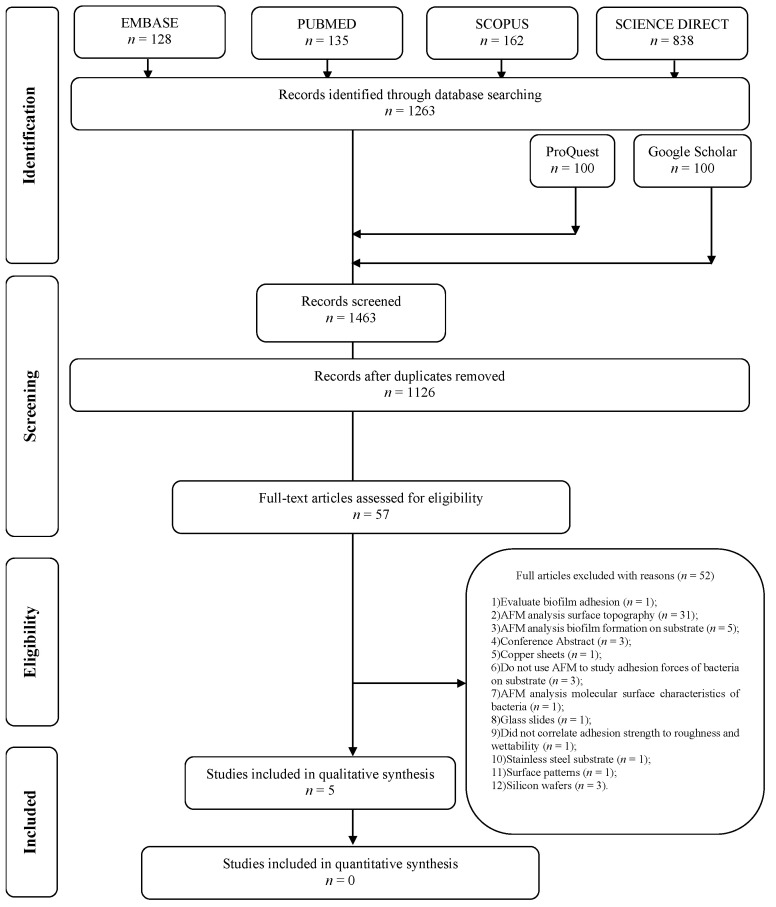
Flow diagram of the literature search and selection criteria.

After removing duplicates, 1126 articles were screened according to the title and abstract, of which 57 were chosen for full reading. Of these, five met the eligibility criteria (Table 1), all experimental in vitro studies that evaluated the adhesion strength of bacteria to titanium surfaces using atomic force microscopy and were thus included in the systematic review, so fifty-two were excluded (Table S1). The article selection process is shown in Figure 1.

### 2.2. Qualitative Assessment of the Studies

Aguayo et al., 2015 [1], and Aguayo et al., 2016 [14], demonstrated that the adhesion strength of *S. aureus* [1] and *S. sanguinis* [14] on slightly hydrophilic Ti of average roughness (Ra) (<1 µm) increases with time, in addition to the hydrophilicity favoring the formation of hydrogen bonds in the bacterium on the substrate.

An et al., 2017 [48], and Du et al., 2022 [49], demonstrated that treatment with poly(ethylene glycol) (PEG) [48] and laser-induced periodic surface structure (LIPSS) [49] increased the roughness and hydrophilicity of the Ti substrate [48,49], Zr43.3Cu27.8Ni15.2Al9.1Ti4.6 (Zr-BMG) [49], stainless steel (316L) [49], and titanium alloy (TC4) [49] and consequently reduced the bacterial adhesion strength of *S. aureus* [48,49] and *E. coli* [49]. As Alam and Balani, 2017 [15], demonstrated, the greater the roughness and hydrophilicity of the Ti-6Al-4V substrate, the greater the adhesion strength of *S. aureus*.

### 2.3. Risk of Bias

The risk of bias in the studies was analyzed as previously performed by Sarkis-Onofre et al., 2014 [50], according to the description of essential parameters to be explored: clarity in the materials section; clarity in the methodology section; roughness, wettability, and bacterial adhesion strength evaluated by a reliable method; sufficient detail to allow replication; and clarity in the results.

The studies by Aguayo et al., 2015 [1], Aguayo et al., 2016 [14], and Alam and Balani, 2017 [15], showed a low risk of bias. The research by An et al., 2017 [48], and Du et al., 2022 [49], displayed a moderate risk of bias.

In the studies by An et al., 2017 [48], and Du et al., 2022 [49], the risk of bias was increased in terms of methodology clarity, as they did not specify the time taken to evaluate the maximum strength of bacterial adhesion to the substrate.

The studies by Alam and Balani, 2017 [15], An et al., 2017 [48], and Du et al., 2022 [49], showed an increased risk of bias for the clarity parameter in the results because they did not state at which point the maximum force of bacterial adhesion to the substrate occurred. In addition, the study by Du et al., 2022 [49], did not show clarity in the results as it did not report the roughness value of all evaluated groups (Figures S1 and S2).

### 2.4. Meta-Analysis

A statistical analysis was not performed due to the heterogeneity of the substrates, surface treatments, methods of evaluating roughness and wettability, and bacteria expressed in Table 2.

## 3. Discussion

The leading cause of the failure of implant-supported rehabilitation is peri-implant infection, and this remains the main reason for patient dissatisfaction with treatment costs needed to contain the infection [1,2,3,4,5,6,7,8,9,10]. The strategies for using systemic antibiotics and antimicrobial surface treatments in implants through ions, such as silver (Ag), copper (Cu), and zinc (Zn), or polymers from the slow release of antibiotics, which still have limitations, such as bacterial resistance, rapid drug release, and even toxicity [3,4,5,6,7,8,9,51,52,53,54,55,56]. Therefore, the search to understand the factors that determine bacterial adhesion to implant surfaces is configured as the way to determine strategies to prevent such contamination and thus increase the survival of implant-supported rehabilitation.

Bacterial adhesion to the implant depends on (1) the physicochemical properties of the material, roughness, wettability, and chemical composition; (2) the intrinsic properties of bacteria, such as their appendages, electrical charge, and hydrophilicity; and (3) the properties of the environment, temperature, pH, antibiotic type, exposure period, and bacterial concentration [57]. Despite the literature [1,10,14,15,16,17,18,19,20,36,57] reporting that the physicochemical properties of the surface influence bacterial adhesion, the mechanisms that regulate this adhesion are still uncertain and contradictory. Therefore, this systematic review aimed to evaluate the five [1,14,15,48,49] in vitro articles critically included, investigating the kinetics of bacterial adhesion as a function of wettability and roughness and by quantifying the strength of adhesion by the atomic force microscopy method.

Ti implants and their alloys are the most used metallic materials for biomedical applications, with high success rates above 92%; however, despite their excellent biocompatibility, corrosion resistance, and mechanical performance, their surface does not have intrinsic antimicrobial properties and is thus susceptible to constant microbial challenge [21,58]. As bacterial adhesion and pathogenic biofilm development are etiological factors of mucositis and peri-implantitis, researchers try to prevent and eradicate this bacterial colonization through surface treatments on Ti and its alloys. However, most of these aim to prevent this colonization with antimicrobial nanomaterials which can cause cytotoxic reactions and bacterial resistance [21,58].

Thus, this systematic review aimed to evaluate the quantification of bacterial adhesion through atomic force microscopy because this is the most efficient technique for studying cell–biomaterial interactions for results at the atomic level [39], which allows a more realistic correlation of how the physical properties, i.e., roughness and wettability, interfere with bacterial adhesion for the development of anti-biofilm surfaces that regulate these properties without the need for antimicrobial agents. The evaluated substrates were Ti [1,14,48] and Ti-6Al-4V [15,49]. When correlating the bacterial adhesion strength with the physical properties, roughness and wettability can infer that the results were discrepant from the literature in terms of these properties.

When analyzing roughness, surfaces with greater roughness [48,49] did not promote the most bacterial adhesion, as inferred in the literature [27,28,29,30,59,60], which suggests that the morphology of peaks and valleys predictive of roughness interferes more than the raw roughness value by reducing areas favorable to adhesion. While for wettability, the adaptive capacity of bacteria allows them to develop mechanisms such as hydrogen bonds to adhere to hydrophilic surfaces even if they are hydrophobic [1,14].

### 3.1. Wettability

Surface wettability influences bacterial adhesion and consequent biofilm formation. Nonetheless, the system is complex and depends on the bacterial species and strain in question and its compositional characteristics, e.g., the surface topography to be evaluated, and the environment [36,57,58]. The surfaces can be classified according to the contact angle of the liquid, with the surface considered as superhydrophilic < 5°, hydrophilic ≤ 90°, hydrophobic > 90°, and superhydrophobic > 150° [36,61,62,63].

Gittens et al., 2014 [36], in their review on the influence of the wettability of dental implants on biological activities, generally inferred that hydrophilic bacterial strains adhere more to hydrophilic surfaces, while hydrophobic ones adhere more to hydrophobic surfaces. The bacterial preference for surfaces of the same hydrophilicity was not observed in the study by Alam and Balani, 2017 [15], in which the bacteria *S. aureus*, considered hydrophobic [10,36], showed the highest adhesion force in the most hydrophilic sample. The same fact has been observed in the studies by Aguayo et al., 2015 [1], and Aguayo et al., 2016 [14], where the hydrophobic bacteria *S. aureus* [1,10,36] and *S. sanguinis* [10,14,36,61,64] showed higher adhesion strength over time on slightly hydrophilic titanium surfaces. According to the authors, this is due to the bacteria forming unspecific bonds with the surface and hydrogen bonds.

Thus, the present review can infer that despite the literature reporting the bacterial preference for surfaces of the same hydrophilicity, bacteria can develop survival mechanisms such as hydrogen bonds, as hydrophobic bacteria adhere to hydrophilic surfaces. This fact is justified by the bacterial constitution of 70% in water, enabling nonspecific bonding and hydrogen bonding with a hydrophilic surface [1,14,65].

### 3.2. Roughness

Regarding the surface roughness, its measurement can be given through the value of Ra, the general average in micrometers of the topography in the two-dimensional vertical direction, obtained through laser profilometry, but it does not differentiate linear and circular features, such as scratches and holes, as these are generally nanometric [14,59,62]. In this context, the AFM presents a greater definition for the three-dimensional nanometric analysis as the most representative roughness scale to which bacteria are exposed [14,63,66]. It should be noted that for the biological area, the Ra parameter is the most used [14,63,66].

**Figure 2:**
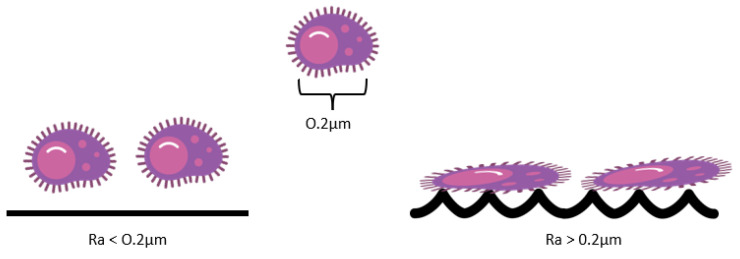
Schematic representation of the influence of the Ra roughness parameter on bacterial adhesion.

According to the literature, surfaces with Ra below 0.2 μm do not promote bacterial adhesion, as the bacterial cell wall cannot detect the topographies of smaller dimensions than the bacteria. In contrast, surfaces with Ra above 150μm are the maximum limit and provide statistical differences in bacterial adhesion (Figure 2) [18,60,67,68]. The bacterial preference for sites that maximize the adhesion area, such as irregularities, grooves, and crevices, produced during surface fabrication, square corners, and convex walls, has been highlighted [57,69,70].

According to the review by Yang et al., 2022 [30], bacterial adhesion on smooth surfaces depends on the material’s physical-chemical properties, while nanometric surfaces are anti-adhesive and micrometric surfaces favor adhesion. Despite the literature [27,28,29,30,59,60] reporting that bacteria prefer to adhere to rougher surfaces, the studies by An et al., 2017 [48], and Du et al., 2022 [49], disagree. According to Ge et al., 2019 [71], the increase in roughness reduced bacterial adhesion strength, possibly due to the surface treatment potentiostat anodization of titanium for nanotube formation [48] or lasers [49], which limit the contact area available for bacterial adhesion. In the study by An et al., 2017 [48], potentiostat anodization surface treatment by inducing an increase in the size of the nanotubes formed on the surface reduces the available contact area for bacteria to adhere to the surface. Consequently, it weakens the bacteria’s biofouling interactions (adhesion and friction) on the surface. The laser treatment of Du et al., 2022 [49], decreased the contact area available for bacterial adhesion by increasing peak height and decreasing the distance between valleys, thus affecting adhesion and restricting bacterial cell motility.

It should be noted that for clinical conditions, rough implants obtained by additive manufacturing have become advantageous as they allow a greater diffusion of biological fluids and nutrients between their pores, which favors adhesion, proliferation, and osteoblastic maturation and optimizes mechanical function by enabling the production of implants with an elastic modulus closer to that of bone tissue [72,73,74].

Unlike the selection of the implant body, the roughness of the transgingival portion of the implant is the subject of questioning. At the same time, some authors prefer smooth transgingival surfaces because they believe that they reduce bacterial adhesion and the consequent risk of peri-implant inflammation [75,76,77], while other authors believe that a rough surface promotes more excellent epithelial adhesion on the abutment, which induces greater protection against the bacterial invasion, which reduces the chances of peri-implantitis [78,79,80,81,82]. It should be noted that the lack of consensus in the literature regarding the selection of the abutment motivates the development of controlled clinical studies with a long follow-up period for a better inference of the causality between the roughness of the abutment and peri-implantitis.

### 3.3. Survival Mechanism

The physical-chemical properties, namely roughness and wettability, influence bacterial adhesion in the first contact phase [1,14,15,48,49]. Because it acts as a predisposing factor to bacterial adhesion, roughness, depending on the morphology of peaks and valleys, allows an increase in the area favorable to adhesion, and wettability acts through the hydrophilic affinity mechanism so that the hydrophilic surfaces of the bacteria can more easily adhere [1,14,15,48,49].

However, another critical parameter to consider is the AFM analysis time evaluated in the studies by Aguayo et al., 2015 [1], Aguayo et al., 2016 [14], and Alam and Balani, 2017 [15]. As the contact time of the bacteria on the surface increases, the resulting force of adhesion increases, regulated by the specific and nonspecific forces of the bacteria on the substrate, which favors the proliferation of bacterial colonies and consequent territorial expansion [1,14,15]. In addition, temporal analysis allows researchers to identify bacterial adaptation mechanisms and possible lethal interactions [1,14,15]. In accordance with Lüdecke et al., 2016 [35], who demonstrated the importance of evaluating bacterial adhesion in time-dependent studies because although the estimated surface was considered anti-adhesive because it was nanometric, there was indeed the formation of bacterial colonies of *S. aureus* as time passed.

The development of antibacterial surfaces through the observation of the biofouling, adhesion, and friction interactions of bacteria in the five [1,14,15,48,49] studies included in this systematic review provided an answer to the question “What biophysical factors should be considered when analyzing the bacterial adhesion strength on titanium surfaces and its alloys for implants quantified by atomic force microscopy?” and infers that the variation of wettability and roughness interfere at first in adhesion strength quantified by atomic force microscopy; however, bacteria develop time-dependent survival mechanisms, as Kreve and Reis, 2021 [20], elucidated. Hence, the development of antifouling surfaces that inhibit the physiological pathways of bacterial adaptation is a strategy that will allow the prevention of peri-implantitis.

According to this review, the development of adaptation mechanisms for the survival of bacteria on titanium surfaces for dental implants is primarily responsible for the conflicting results regarding wettability and the bacterial preference for surfaces of the same hydrophilicity and roughness. The main limitation of this systematic review is the lack of understanding of the adaptation mechanisms developed by bacteria to adapt to the substrates. Still, it is justified that these biomolecular mechanisms were obscure because they were not the focus of the present review.

In this systematic review, the physical properties, roughness, and wettability were correlated with the bacterial adhesion strength on Ti substrates for biomedical applications. In addition to these, the authors of this systematic review would like to highlight the importance of also considering the electrostatic condition of the surface, which significantly influences bacterial adhesion and may be a plausible justification for the discrepant results regarding roughness and wettability found in this study, as demonstrated by the review by Kreve and Reis, 2021 [10]. Therefore, the surface properties, electrical potential, chemical composition, wettability, and roughness interact synergistically with the bacterial strain.

### 3.4. Clinical Implications

The installation of dental implants in the oral cavity is challenging because although this surgery is performed with an antiseptic methodology to avoid contamination, the oral environment is polymicrobial with roughly a thousand bacterial species detectable by sequencing the 16sRNA gene, so there is a possibility that infection could be caused microorganisms present in saliva [21,83,84]. On this subject, Johansson et al., 2017 [83], carried out a systematic review to verify the existence of scientific evidence that contamination during dental implant surgery influenced osseointegration and clinical success as it affects orthopedic surgeries. However, the findings of the results were insufficient for a definitive conclusion, and the authors suggest the clinician be cautious in order to preventing infections, corroborating the results of Albrekston et al., 1981 [85], which demonstrate that a clinician’s skill during surgery is a predictive factor of success because it influences surgical time.

Another factor to be considered that predisposes to contamination in dental implants is the histology of the peri-implant tissue in the transmucosal region, as it is made up of collagen fibers arranged in parallel, unlike the dental area, which is perpendicular; thus, the adhesive union in the peri-implant transmucosal region is inferior and considered an adaptation and not a fixation as when it occurs in the tooth [86,87,88]. Therefore, when considering the transmucosal region, implants can be positioned at tissue and bone level; scientific evidence demonstrates that their position is associated with biological complications, so implants with a smaller mucosal tunnel have lower rates of complications, crestal bone loss, and peri-implantitis, as demonstrated by the study by Chan et al., 2019 [89], and Rokn et al., 2017 [90], for enabling more effective oral hygiene.

Thus, as the process of bacterial pathogenesis in implants is multifactorial, involving the physicochemical properties of the biomaterial, bacterial strain type, environmental conditions, and other causes that are present even when trying to avoid contamination when installing the implant and prosthetic component [57,83,85,89,90,91], the authors of this review suggest that the clinician, when aware of the possibility of contamination, be cautious with strict antisepsis measures in their clinical practice and instruct the patient on the risk of biological complications and that, in an attempt to avoid such problems, daily biofilm control and regular appointments are necessary.

## 4. Materials and Methods

Detailed in the Supplementary Materials.

## 5. Conclusions

(1) Current literature shows the preference of bacteria in adhering to the surfaces of the same hydrophilicity. However, this is contradicted by the present systematic review, which showed that this might not be the case, since hydrophobic bacteria developed hydrogen bonds and adhered to hydrophilic surfaces.
(2) The application of surface treatments, such as potentiostat anodization and lasers, that induce the reduction of areas favorable for bacterial adhesion interferes more in the formation of biofilm than the surface roughness.
(3) Bacterial colonization should be evaluated in time-dependent studies as they develop adaptation mechanisms related to time, which are not examined in this review. Furthermore, the electrostatic condition of the surface is a property that should be highlighted.
(4) For clinical conditions, the literature demonstrates the following: 1. Positioning the implant at the tissue level is recommended as it allows for better hygiene; 2. There is still no consensus on the ideal roughness for the transmucosal region of the implant; 3. Printed implants allow better adhesion and osteoblastic proliferation and control of their printing parameters for mechanical biocompatibility with bone tissue; and 4. The oral cavity is a polymicrobial area so implant contamination can occur during its installation; thus, the clinician must be aware of the possibility of contamination and avoid it by using strict antisepsis methods and instruct the patient on the need for regular consultations and on how to sanitize.

## Figures and Tables

**Table 1:** Included articles.

Author, Year	Title
Aguayo et al., 2015 [1]	Nanoadhesion of *Staphylococcus aureus* onto Titanium Implant Surfaces
Aguayo et al., 2016 [14]	Probing the nanoadhesion of *Streptococcus sanguinis* to titanium implant surfaces by atomic force microscopy
Alam and Balani 2017 [15]	Adhesion force of *Staphylococcus aureus* on various biomaterial surfaces
An et al., 2017 [48]	Adhesion and friction forces in biofouling attachments to nanotubeand PEG-patterned TiO_2_ surfaces
Du et al., 2022 [49]	Antibacterial Performance of Zr-BMG, Stainless Steel, and Titanium Alloy with Laser-Induced Periodic Surface Structures

**Table 2:** Characteristics of the included studies.

Author, Year	Population	Wettability Assessment Method	Wettability Results	Roughness Assessment Method	Roughness Results	Bacteria	Bacterial Adhesion Strength by AFM Result	Conclusion
Aguayo et al., 2015 [1]	Ti Sample= Ti	Optical contact angle meter (CAM 200, KSV Instruments, Biolin Scientific, MD, USA)	67.0 ± 5°	Profilometry (Proscan 1000, Scantron Ltd., Somerset, UK)	Ra 0.61 µm	*S. aureus*	0 s = −0.27 ± 0.30 nN 60 s = −9.15 ± 0.78 nN	The binding strength of *S. aureus* on Ti increases with time. The slightly hydrophilic surface favored the formation of hydrogen bonds between the bacterium and the substrate. The roughness characterizes the surface as smooth Ra < 1 µm.
Aguayo et al., 2016 [14]	Ti Sample = Ti	Optical contact angle meter (KSV Instruments, CAM 200, Monroe, CT, USA)	67.0° ± 5.0°	Conventional profilometry (Proscan 1000, Scantron, Somerset, UK) and AFM profilometry (Gwyddion 2.31 software, *n* = 9256 × 256 pixel scans)	Ra CP= 0.61 ± 0.01 μm and AFM = 0.17 ± 0.02 μm	*S. sanguinis*	0 s = 0.32 ± 0.00 nN 1 s = 1.07 ± 0.06 nN 60 s = 4.85 ± 0.56 nN	The binding strength of *S. sanguinis* on Ti increases with time. The slightly hydrophilic surface that favored the formation of hydrogen bonds between the bacterium and the substrate. The roughness characterizes the surface as smooth Ra < 1 µm.
Alam and Balani 2017 [15]	G1 = Ti-6Al-4V; G2 = UHMWPE; G3 = SS; G4 = HA.	contact angle goniometer (Dataphysics Contact Angle System OCA)	Ti-6Al-4V = 68.8 ± 5.6 UHMWPE= 81.9 ± 2.3 SS= 48.7 ± 1.9 HA= 94.9 ± 1.2	AFM by Nanoscope Analysis (Bruker, version 1.40)	Ra Ti-6Al-4V = 289 nm UHMWPE = 70 nm SS = 219 nm HA = 229 nm	*S. aureus*	0 a 10 s Maximum adhesion strength = Ti-6Al-4V = 11.12 ± 1.07 UHMWPE = 4.10 ± 0.65 SS = 15.21 ± 1.41 HA = 7.66 ± 0.67	Adhesion strength increased with time; the greater the roughness and wettability, the greater the adhesion force.
An et al., 2017 [48]	Ti Groups = G1 = DT; G2 = TN20; G3 = TN80; G4 = DT-P; G5 = TN20-P; G6 = TN80-P.	Contact angles by a DSA100 (Kruss, Germany)	DT = 96.1 ± 2.6°; TN20 = 78 ± 0.9°; TN80 = 19.5 ± 0.7°; DT-P = 68.8 ± 1.5°; TN20-P, 15.2 ± 0.1°; TN80-P, 21.2 ± 0.6°.	AFM (AFM, Dimension Icon, Bruker, USA)	RMS DT = 1.06 nm; TN20 = 19.0 nm; TN80 = 29.2 nm; DT-P = 5.13 nm; TN20-P = 28.1 nm; TN80-P = 41.2 nm.	*S. aureus*	Maximum adhesion strength = = DT = 172.6 ± 6.12 nN; TN20 = 31.3 ± 6.1 nN; TN80 = 32.4 ± 1.89 nN; DT-P = 149.3 ± 6.22 nN; TN20-P = 72.5 ± 3.43 nN; TN80-P = 26.4 ± 3.75 nN.	The addition of PEG increased the hydrophilicity and roughness. However, it decreased bacterial adhesion and adhesion strength. It is noteworthy that the presence of nanotubes decreased the surface area and increased the hydrophilicity.
Du et al., 2022 [49]	G1 = Zr-BMG; G2 = 316L; G3 = TC4; G4 = Zr-BMG-LIPSS; G5 = 316L-LIPSS; G6 = TC4-LIPSS.	Contact angle goniometer (OSA-200)	Zr-BMG= 73.6 ± 0.7°; 316L = 72.9 ± 0.5°; TC4 = 64.3 ± 0.7°; Zr-BMG-LIPSS = 28.3 ± 1.4°; 316L-LIPSS = 25.2 ± 0.9°; TC4-LIPSS = 20.6 ± 1.1°.	AFM (Bruker fast scan)	Ra Zr-BMG = NS; 316L = NS; TC4 = NS; Zr-BMG-LIPSS = 68.3 ± 9.1 nm; 316L-LIPSS = 54.7 ± 7.3 nm; TC4-LIPSS = 118 ± 10.6 nm.	*E. coli* and *S. aureus*.	*E. coli* Zr-BMG = 4.47 ± 0.73 nN; 316L = 4.37 ± 0.73 nN; TC4 = 4.87 ± 0.63 nN; Zr-BMG-LIPSS = 2.51 ± 0.59 nN; 316L-LIPSS = 2.61 ± 0.79 nN; TC4-LIPSS = 2.47 ± 0.73 nN. *S. aureus* Zr-BMG = 4.05 ± 0.45 nN; 316L = 3.98 ± 0.62 nN; TC4 = 4.74 ± 0.56 nN; Zr-BMG-LIPSS = 0.88 ± 0.52 nN; 316L-LIPSS = 0.93 ± 0.47 nN; TC4-LIPSS = 1.06 ± 0.44 nN.	The LIPSS treatment reduced the bacterial adhesion strength for *E. coli* and *S. aureus* in all samples and increased the roughness and wettability of the samples.

316L, stainless steel; AFM, atomic force microscopy; CP, conventional profilometry; DT, the dense TiO_2_ substrate; DT-P, PEG onto DT; HA, hydroxyapatite; LIPSS, a laser-induced periodic surface structure; NS, not specified; PEG, poly(ethylene glycol); RMS, root mean square; S, seconds; SS, stainless steel; TC4, titanium alloy; TN20, nanotubular TiO_2_ surface with pore size of 20 nm; TN20-P, PEG onto TN20; TN80, nanotubular TiO_2_ nanotubes of about 80 nm; TN80-P, PEG onto TN80; UHMWPE, ultra-high molecular weight poly ethylene; Zr-BMG, Zr43.3Cu27.8Ni15.2Al9.1Ti4.6.

## Data Availability

Not applicable.

## Supplementary Materials

The following supporting information can be downloaded at: https://www.mdpi.com/article/10.3390/antibiotics12060994/s1, Materials and Methods; Figure S1: risk of bias of this systematic review; Figure S2: Risk of bias per study included; Supplemental Table S1: Databases and search strategy; Supplemental Table S2: Excluded articles and reasons for exclusion.

## References

[1] Aguayo S., Donos N., Spratt D., Bozec L. (2015). Nanoadhesion of Staphylococcus aureus onto titanium implant surfaces. J. Dent. Res..

[2] Barbour M.E., O’Sullivan D.J., Jenkinson H.F., Jagger D.C. (2007). The effects of polishing methods on surface morphology, roughness and bacterial colonisation of titanium abutments. J. Mater. Sci. Mater. Med..

[3] Beltrán-Partida E., Valdez-Salas B., Escamilla A., Moreno-Ulloa A., Burtseva L., Valdez-Salas E., Curiel Alvarez M., Nedev N. (2015). The Promotion of Antibacterial Effects of Ti6Al4V Alloy Modified with TiO_2_ Nanotubes Using a Superoxidized Solution. J. Nanomater..

[4] Ou K.L., Weng C.C., Lin Y.H., Huang M.S. (2017). A promising of alloying modified beta-type Titanium-Niobium implant for biomedical applications: Microstructural characteristics, in vitro biocompatibility and antibacterial performance. J. Alloys Compd..

[5] Kazek-Kęsik A., Nosol A., Płonka J., Śmiga-Matuszowicz M., Gołda-Cępa M., Krok-Borkowicz M., Brzychczy-Włoch M., Pamuła E., Simka W. (2019). PLGA-amoxicillin-loaded layer formed on anodized Ti alloy as a hybrid material for dental implant applications. Mater. Sci. Eng. C.

[6] Walter M.S., Frank M.J., Satué M., Monjo M., Rønold H.J., Lyngstadaas S.P., Haugen H.J. (2014). Bioactive implant surface with electrochemically bound doxycycline promotes bone formation markers in vitro and in vivo. Dent. Mater..

[7] Kazek-Kęsik A., Nosol A., Płonka J., Śmiga-Matuszowicz M., Student S., Brzychczy-Włoch M., Krok-Borkowicz M., Pamuła E., Simka W. (2020). Physico-chemical and biological evaluation of doxycycline loaded into hybrid oxide-polymer layer on Ti–Mo alloy. Bioact. Mater..

[8] Rangel A.L.R., Falentin-Daudré C., da Silva Pimentel B.N.A., Vergani C.E., Migonney V., Alves Claro A.P.R. (2020). Nanostructured titanium alloy surfaces for enhanced osteoblast response: A combination of morphology and chemistry. Surf. Coat. Technol..

[9] Kazek-Kęsik A., Jaworska J., Krok-Borkowicz M., Gołda-Cępa M., Pastusiak M., Brzychczy-Włoch M., Pamuła E., Kotarba A., Simka W. (2016). Hybrid oxide-polymer layer formed on Ti-15Mo alloy surface enhancing antibacterial and osseointegration functions. Surf. Coat. Technol..

[10] Kreve S., Cândido dos Reis A. (2021). Influence of the electrostatic condition of the titanium surface on bacterial adhesion: A systematic review. J. Prosthet. Dent..

[11] Ahimou F., Semmens M.J., Novak P.J., Haugstad G. (2007). Biofilm cohesiveness measurement using a novel atomic force microscopy methodology. Appl. Environ. Microbiol..

[12] Matos A.O., Ricomini-Filho A.P., Beline T., Ogawa E.S., Costa-Oliveira B.E., de Almeida A.B., Nociti Junior F.H., Rangel E.C., da Cruz N.C., Sukotjo C. (2017). Three-species biofilm model onto plasma-treated titanium implant surface. Colloids Surf. B Biointerfaces.

[13] Boshkovikj V., Fluke C.J., Crawford R.J., Ivanova E.P. (2014). Three-dimensional visualization of nanostructured surfaces and bacterial attachment using Autodesk^®^ Maya^®^. Sci. Rep..

[14] Aguayo S., Donos N., Spratt D., Bozec L. (2016). Probing the nanoadhesion of Streptococcus sanguinis to titanium implant surfaces by atomic force microscopy. Int. J. Nanomed..

[15] Alam F., Balani K. (2017). Adhesion force of *staphylococcus aureus* on various biomaterial surfaces. J. Mech. Behav. Biomed. Mater..

[16] Caro-Lara L., Ramos-Moore E., Vargas I.T., Walczak M., Fuentes C., Gómez A.V., Barrera N.P., Castillo J., Pizarro G. (2021). Initial adhesion suppression of biofilm-forming and copper-tolerant bacterium *Variovorax* sp. on laser microtextured copper surfaces. Colloids Surf. B Biointerfaces.

[17] Gadenne V., Lebrun L., Jouenne T., Thebault P. (2013). Antiadhesive activity of *ulvan polysaccharides* covalently immobilized onto titanium surface. Colloids Surf. B Biointerfaces.

[18] Golda-Cepa M., Brzychczy-Wloch M., Engvall K., Aminlashgari N., Hakkarainen M., Kotarba A. (2015). Microbiological investigations of oxygen plasma treated parylene C surfaces for metal implant coating. Mater. Sci. Eng. C.

[19] Na C., McNamara C.J., Konkol N.R., Bearce K.A., Mitchell R., Martin S.T. (2010). The use of force-volume microscopy to examine bacterial attachment to titanium surfaces. Ann. Microbiol..

[20] Kreve S., Reis A.C.D. (2021). Bacterial adhesion to biomaterials: What regulates this attachment?. A Rev. Jpn. Dent. Sci. Rev..

[21] Souza J.G.S., Bertolini M.M., Costa R.C., Nagay B.E., Dongari-Bagtzoglou A., Barão V.A.R. (2021). Targeting implant-associated infections: Titanium surface loaded with antimicrobial. iScience.

[22] Bürgers R., Morsczeck C., Felthaus O., Gosau M., Beck H.C., Reichert T.E. (2018). Induced surface proteins of *Streptococcus epidermidis* adhering to titanium implant substrata. Clin. Oral Investig..

[23] Siddiqui D.A., Fidai A.B., Natarajan S.G., Rodrigues D.C. (2022). Succession of oral bacterial colonizers on dental implant materials: An in vitro biofilm model. Dent. Mater..

[24] Fürst M.M., Salvi G.E., Lang N.P., Persson G.R. (2007). Bacterial colonization immediately after installation on oral titanium implants. Clin. Oral Implant. Res..

[25] Rickard A.H., Gilbert P., High N.J., Kolenbrander P.E., Handley P.S. (2003). Bacterial coaggregation: An integral process in the development of multi-species biofilms. Trends Microbiol..

[26] Wake N., Asahi Y., Noiri Y., Hayashi M., Motooka D., Nakamura S., Gotoh K., Miura J., Machi H., Iida T. (2016). Temporal dynamics of bacterial microbiota in the human oral cavity determined using an in situ model of dental biofilms. Npj Biofilms Microbiomes.

[27] Kaliaraj G.S., Bavanilathamuthiah M., Kirubaharan K., Ramachandran D., Dharini T., Viswanathan K., Vishwakarma V. (2016). Bio-inspired YSZ coated titanium by EB-PVD for biomedical applications. Surf. Coat. Technol..

[28] Azelmad K., Hamadi F., Mimouni R., Amzil K., Latrache H., Mabrouki M., El Boulani A. (2017). Adhesion of *Staphylococcus aureus* and *Staphylococcus xylosus* to materials commonly found in catering and domestic kitchens. Food Control.

[29] Yoda I., Koseki H., Tomita M., Shida T., Horiuchi H., Sakoda H., Osaki M. (2014). Effect of surface roughness of biomaterials on *Staphylococcus epidermidis* adhesion. BMC Microbiol..

[30] Yang K., Shi J., Wang L., Chen Y., Liang C., Yang L., Wang L.N. (2022). Bacterial anti-adhesion surface design: Surface patterning, roughness and wettability: A review. J. Mater. Sci. Technol..

[31] Vanhaecke E., Remon J.P., Moors M., Raes F., De Rudder D., Van Peteghem A. (1990). Kinetics of Pseudomonas aeruginosa adhesion to 304 and 316-L stainless steel: Role of cell surface hydrophobicity. Appl. Environ. Microbiol..

[32] Braceras I., Pacha-Olivenza M.A., Calzado-Martín A., Multigner M., Vera C., Broncano L.L., Gallardo-Moreno A.M., González-Carrasco J.L., Vilaboa N., González-Martín M.L. (2014). Decrease of Staphylococcal adhesion on surgical stainless steel after Si ion implantation. Appl. Surf. Sci..

[33] Wassmann T., Kreis S., Behr M., Buergers R. (2017). The influence of surface texture and wettability on initial bacterial adhesion on titanium and zirconium oxide dental implants. Int. J. Implant. Dent..

[34] Wu Y., Zitelli J.P., TenHuisen K.S., Yu X., Libera M.R. (2011). Differential response of *Staphylococci* and *osteoblasts* to varying titanium surface roughness. Biomaterials.

[35] Lüdecke C., Roth M., Yu W., Horn U., Bossert J., Jandt K.D. (2016). Nanorough titanium surfaces reduce adhesion of *Escherichia coli* and *Staphylococcus aureus* via nano adhesion points. Colloids Surf. B Biointerfaces.

[36] Gittens R.A., Scheideler L., Rupp F., Hyzy S.L., Geis-Gerstorfer J., Schwartz Z., Boyan B.D. (2014). A review on the wettability of dental implant surfaces II: Biological and clinical aspects. Acta Biomater..

[37] Qi M., Gong X., Wu B., Zhang G. (2017). Landing Dynamics of Swimming Bacteria on a Polymeric Surface: Effect of Surface Properties. Langmuir.

[38] Boks N.P., Busscher H.J., Van Der Mei H.C., Norde W. (2008). Hydrophobic Surfaces Using Atomic Force Microscopy. Langmuir.

[39] Alam F., Kumar S., Varadarajan K.M. (2019). Quantification of Adhesion Force of Bacteria on the Surface of Biomaterials: Techniques and Assays. ACS Biomater. Sci. Eng..

[40] Viljoen A., Mignolet J., Viela F., Mathelié-Guinlet M., Dufrêne Y.F. (2020). How Microbes Use Force to Control Adhesion. J. Bacteriol..

[41] Sugimoto Y., Pou P., Abe M., Jelinek P., Pérez R., Morita S., Custance O. (2007). Chemical identification of individual surface atoms by atomic force microscopy. Nature.

[42] Giessibl F.J. (2003). Advances in atomic force microscopy. Rev. Mod. Phys..

[43] Berne C., Ellison C.K., Ducret A., Brun Y.V. (2018). Bacterial adhesion at the single-cell level. Nat. Rev. Microbiol..

[44] Ren Y., Wang C., Chen Z., Allan E., van der Mei H.C., Busscher H.J. (2018). Emergent heterogeneous microenvironments in biofilms: Substratum surface heterogeneity and bacterial adhesion force-sensing. FEMS Microbiol. Rev..

[45] Milles L.F., Gaub H.E. (2020). Extreme mechanical stability in protein complexes. Curr. Opin. Struct. Biol..

[46] Grzeszczuk Z., Rosillo A., Owens Ó., Bhattacharjee S. (2020). Atomic Force Microscopy (AFM) As a Surface Mapping Tool in Microorganisms Resistant Toward Antimicrobials: A Mini-Review. Front. Pharmacol..

[47] James S.A., Hilal N., Wright C.J. (2017). Atomic force microscopy studies of bioprocess engineering surfaces—Imaging, interactions and mechanical properties mediating bacterial adhesion. Biotechnol. J..

[48] An R., Dong Y., Zhu J., Rao C. (2017). Adhesion and friction forces in biofouling attachments to nanotube- and PEG-patterned TiO_2_ surfaces. Colloids Surf. B Biointerfaces.

[49] Du C., Wang C., Zhang T., Zheng L. (2022). Antibacterial Performance of Zr-BMG, Stainless Steel, and Titanium Alloy with Laser-Induced Periodic Surface Structures. ACS Appl. Bio. Mater..

[50] Sarkis-Onofre R., Skupien J.A., Cenci M.S., Moraes R.R., Pereira-Cenci T. (2014). The role of resin cement on bond strength of glass-fiber posts luted into root canals: A systematic review and metaanalysis of in vitro studies. Oper. Dent..

[51] Cunha A., Elie A.M., Plawinski L., Serro A.P., Botelho Do Rego A.M., Almeida A., Urdaci M.C., Durrieu M.C., Vilar R. (2016). Femtosecond laser surface texturing of titanium as a method to reduce the adhesion of Staphylococcus aureus and biofilm formation. Appl. Surf. Sci..

[52] Dong Y., Ye H., Liu Y., Xu L., Wu Z., Hu X., Ma J., Pathak J.L., Liu J., Wu G. (2017). pH dependent silver nanoparticles releasing titanium implant: A novel therapeutic approach to control peri-implant infection. Colloids Surf. B Biointerfaces.

[53] Bartmanski M., Cieslik B., Glodowska J., Kalka P., Pawlowski L., Pieper M., Zielinski A. (2017). Electrophoretic deposition (EPD) of nanohydroxyapatite—Nanosilver coatings on Ti13Zr13Nb alloy. Ceram. Int..

[54] Sedelnikova M.B., Komarova E.G., Sharkeev Y.P., Ugodchikova A.V., Mushtovatova L.S., Karpova M.R., Sheikin V.V., Litvinova L.S., Khlusov I.A. (2019). Zn-, Cu- or Ag-incorporated micro-arc coatings on titanium alloys: Properties and behavior in synthetic biological media. Surf. Coat. Technol..

[55] Zhang Y., Chu K., He S., Wang B., Zhu W., Ren F. (2020). Fabrication of high strength, antibacterial and biocompatible Ti-5Mo-5Ag alloy for medical and surgical implant applications. Mater. Sci. Eng. C.

[56] Tsao L.C. (2015). Effect of Sn addition on the corrosion behavior of Ti-7Cu-Sn cast alloys for biomedical applications. Mater. Sci. Eng. C.

[57] Santhosh Kumar S., Hiremath S.S., Ramachandran B., Muthuvijayan V. (2019). Effect of Surface Finish on Wettability and Bacterial Adhesion of Micromachined Biomaterials. Biotribology.

[58] Costa R.C., Nagay B.E., Bertolini M., Costa-Oliveira B.E., Sampaio A.A., Retamal-Valdes B., Shibli J.A., Feres M., Barão V.A.R., Souza J.G.S. (2021). Fitting pieces into the puzzle: The impact of titanium-based dental implant surface modifications on bacterial accumulation and polymicrobial infections. Adv. Colloid Interface Sci..

[59] Ferraris S., Truffa Giachet F., Miola M., Bertone E., Varesano A., Vineis C., Cochis A., Sorrentino R., Rimondini L., Spriano S. (2017). Nanogrooves and keratin nanofibers on titanium surfaces aimed at driving gingival fibroblasts alignment and proliferation without increasing bacterial adhesion. Mater. Sci. Eng. C.

[60] Anselme K., Davidson P., Popa A.M., Giazzon M., Liley M., Ploux L. (2010). The interaction of cells and bacteria with surfaces structured at the nanometre scale. Acta Biomater..

[61] Jain S., Williamson R.S., Marquart M., Janorkar A.V., Griggs J.A., Roach M.D. (2018). Photofunctionalization of anodized titanium surfaces using UVA or UVC light and its effects against Streptococcus sanguinis. J. Biomed. Mater. Res. Part B Appl. Biomater..

[62] Gittens R.A., Olivares-Navarrete R., Cheng A., Anderson D.M., McLachlan T., Stephan I., Geis-Gerstorfer J., Sandhage K.H., Fedorov A.G., Rupp F. (2013). The roles of titanium surface micro/nanotopography and wettability on the differential response of human osteoblast lineage cells. Acta Biomater..

[63] Rupp F., Gittens R.A., Scheideler L., Marmur A., Boyan B.D., Schwartz Z., Geis-Gerstorfer J. (2014). A review on the wettability of dental implant surfaces I: Theoretical and experimental aspects. Acta Biomater..

[64] Verdeguer P., Gil J., Punset M., Manero J.M., Nart J., Vilarrasa J., Ruperez E. (2022). Citric Acid in the Passivation of Titanium Dental Implants: Corrosion Resistance and Bactericide Behavior. Materials.

[65] Lin M.H., Wang Y.H., Kuo C.H., Ou S.F., Huang P.Z., Song T.Y., Chen Y.C., Chen S.T., Wu C.H., Hsueh Y.H. (2021). Hybrid ZnO/chitosan antimicrobial coatings with enhanced mechanical and bioactive properties for titanium implants. Carbohydr. Polym..

[66] Verran J., Packer A., Kelly P.J., Whitehead K.A. (2010). Use of the atomic force microscope to determine the strength of bacterial attachment to grooved surface features. J. Adhes. Sci. Technol..

[67] Petrini M., Giuliani A., Di Campli E., Di Lodovico S., Iezzi G., Piattelli A., D’ercole S. (2020). The bacterial anti-adhesive activity of double-etched titanium (Dae) as a dental implant surface. Int. J. Mol. Sci..

[68] Amoroso P.F., Adams R.J., Waters M.G.J., Williams D.W. (2006). Titanium surface modification and its effect on the adherence of *Porphyromonas gingivalis*: An in vitro study. Clin. Oral Implant. Res..

[69] Vadillo-Rodríguez V., Guerra-García-Mora A.I., Perera-Costa D., Gónzalez-Martín M.L., Fernández-Calderón M.C. (2018). Bacterial response to spatially organized microtopographic surface patterns with nanometer scale roughness. Colloids Surf. B Biointerfaces.

[70] Badihi Hauslich L., Sela M.N., Steinberg D., Rosen G., Kohavi D. (2013). The adhesion of oral bacteria to modified titanium surfaces: Role of plasma proteins and electrostatic forces. Clin. Oral Implant. Res..

[71] Ge X., Ren C., Ding Y., Chen G., Lu X., Wang K., Ren F., Yang M., Wang Z., Li J. (2019). Micro/nano-structured TiO_2_ surface with dual-functional antibacterial effects for biomedical applications. Bioact. Mater..

[72] Matsko A., França R. (2022). Design, manufacturing and clinical outcomes for additively manufactured titanium dental implants: A systematic review. Dent. Rev..

[73] Tunchel S., Blay A., Kolerman R., Mijiritsky E., Shibli J.A. (2016). 3D Printing/Additive Manufacturing Single Titanium Dental Implants: A Prospective Multicenter Study with 3 Years of Follow-Up. Int. J. Dent..

[74] Shibli J.A., Mangano C., Mangano F., Rodrigues J.A., Cassoni A., Bechara K., Ferreia J.D.B., Dottore A.M., Iezzi G., Piattelli A. (2013). Bone-to-Implant Contact Around Immediately Loaded Direct Laser Metal-Forming Transitional Implants in Human Posterior Maxilla. J. Periodontol..

[75] Kloss F.R., Steinmüller-Nethl D., Stigler R.G., Ennemoser T., Rasse M., Hächl O. (2011). In vivo investigation on connective tissue healing to polished surfaces with different surface wettability. Clin. Oral Implant. Res..

[76] Abrahamsson I., Zitzmann N.U., Berglundh T., Linder E., Wennerberg A., Lindhe J. (2002). The mucosal attachment to titanium implants with different surface characteristics: An experimental study in dogs. J. Clin. Periodontol..

[77] Albrektsson T., Canullo L., Cochran D., De Bruyn H. (2016). “Peri-Implantitis”: A Complication of a Foreign Body or a Man-Made “Disease”. Facts and Fiction. Clin. Implant. Dent. Relat. Res..

[78] Schliephake H., Scharnweber D., Dard M., Sewing A., Aref A., Roessler S. (2005). Functionalization of dental implant surfaces using adhesion molecules. J. Biomed. Mater. Res. Part B Appl. Biomater..

[79] Deligianni D.D., Katsala N., Ladas S., Sotiropoulou D., Amedee J., Missirlis Y.F. (2001). Effect of surface roughness of the titanium alloy Ti–6Al–4V on human bone marrow cell response and on protein adsorption. Biomaterials.

[80] Fröjd V., Linderbäck P., Wennerberg A., Chávez de Paz L., Svensäter G., Davies J.R. (2011). Effect of nanoporous TiO_2_ coating and anodized Ca2+ modification of titanium surfaces on early microbial biofilm formation. BMC Oral Health.

[81] Degidi M., Artese L., Piattelli A., Scarano A., Shibli J.A., Piccirilli M., Perrotti V., Iezzi G. (2012). Histological and immunohistochemical evaluation of the peri-implant soft tissues around machined and acid-etched titanium healing abutments: A prospective randomised study. Clin. Oral Investig..

[82] Rompen E., Domken O., Degidi M., Farias Pontes A.E., Piattelli A. (2006). The effect of material characteristics, of surface topography and of implant components and connections on soft tissue integration: A literature review. Clin. Oral Implant. Res..

[83] Johansson K., Jimbo R., Östlund P., Tranæus S., Becktor J.P. (2017). Effects of Bacterial Contamination on Dental Implants during Surgery: A Systematic Review. Implant. Dent..

[84] Mark Welch J.L., Dewhirst F.E., Borisy G.G. (2019). Biogeography of the Oral Microbiome: The Site-Specialist Hypothesis. Annu. Rev. Microbiol..

[85] Albrektsson T., Brånemark P.I., Hansson H.A., Lindström J. (1981). Osseo integrated titanium implants. Requirements for ensuring a long-lasting, direct bone-to-implant anchorage in man. Acta Orthop. Scand..

[86] Guo T., Gulati K., Arora H., Han P., Fournier B., Ivanovski S. (2021). Race to invade: Understanding soft tissue integration at the transmucosal region of titanium dental implants. Dent. Mater..

[87] Ivanovski S., Lee R. (2018). Comparison of peri-implant and periodontal marginal soft tissues in health and disease. Periodontol. 2000.

[88] Branemark P.-I. (1983). Osseointegration and its experimental background. J. Prosthet. Dent..

[89] Chan D., Pelekos G., Ho D., Cortellini P., Tonetti M.S. (2019). The depth of the implant mucosal tunnel modifies the development and resolution of experimental peri-implant mucositis: A case–control study. J. Clin. Periodontol..

[90] Rokn A., Aslroosta H., Akbari S., Najafi H., Zayeri F., Hashemi K. (2017). Prevalence of peri-implantitis in patients not participating in well-designed supportive periodontal treatments: A cross-sectional study. Clin. Oral Implant. Res..

[91] Cheng Y., Feng G., Moraru C.I. (2019). Micro- and Nanotopography Sensitive Bacterial Attachment Mechanisms: A Review. Front. Microbiol..

