# Supplementary material for: Bacterial Adhesion Strength on Titanium Surfaces Quantified by Atomic Force Microscopy: A Systematic Review
Source: Antibiotics (Basel). 2023 Jun 1;12(6):994. doi: 10.3390/antibiotics12060994 (PMC10295333; doi:10.3390/antibiotics12060994)
Supplement: Supplementary file 1 [file antibiotics-12-00994-s001.zip › MATERIAL AND METHODS.pdf]

4. Materials and Methods

4.1 Protocol

This systematic review was prepared according to the Preferred Reporting Items for Systematic Review and Meta Analyzes Protocols (PRISMA) [1] and registered in the Open Science Framework (osf.io/spvmb) to answer the question "What regulates bacterial adhesion strength on titanium or its alloys implant surface quantified by atomic force microscopy?". The acronym PECOS contemplated in this systematic review was: Population=surfaces for implants of Ti and its alloys; Exposure=inherent properties of substrates; Comparison=groups present in the study, if any; Outcome=strength of bacterial adhesion on titanium surfaces employing atomic force microscopy, Studies=in vitro experimental studies.

4.2 Eligibility Criteria

Experimental in vitro studies that evaluated the adhesion strength of bacteria to titanium surfaces using atomic force microscopy were included, without the restriction of time and language. It was excluded: 1) Evaluate biofilm adhesion, 2) AFM analysis surface topography; 3) AFM analysis biofilm formation on a substrate, 4) Conference Abstract; 5) Copper sheets; 6) Do not use AFM to study adhesion forces of bacteria on a substrate; 7) AFM analysis molecular surface characteristics of bacteria; 8) Glass slides; 9) Did not correlate adhesion strength to roughness and wettability; 10) Stainless steel substrate; 11) Surface patterns; 12) silicon wafers. After selection, it was verified which physicochemical properties were present in all selected studies, so the roughness and wettability were chosen for the correlation.

4.3 Search strategy

Embase, PubMed, Scopus, and Science Direct databases were used for the personalized search strategy (Table S2). The EndNote X8 software was used to remove duplicates and the Rayyan web application was used to select articles by title and abstract.

4.4 Selection process

The reviewers J.D.C.T and A.C.R independently evaluated the articles found in two phases according to the eligibility criteria. In the first phase, they selected based on title and abstract, and in the second, they read the entire article. The consensus meeting resolved any disagreements and discrepancies.

4.5 Data tabulation

J.D.C.T and A.C.R tabulated data in a Word spreadsheet independently according to author, year; population; method of assessment wettability; wettability result; method of assessment roughness; roughness result; bacteria; Bacterial adhesion strength by AFM result; and conclusion expressed in Table 2.

2.6 Risk of bias analysis

The risk of bias in the studies was analyzed as previously performed by Sarkis-Onofre et al. [2] according to the description of important parameters to be explored: clarity in the materials section, clarity in the methodology section, roughness, wettability, and bacterial adhesion strength evaluated by a reliable method, sufficient detail to allow replication, clarity in the results. As for the parameters reported, the article is scored with "Y" (yes), if not "N" (no). The risk of bias classification was performed according to the number of parameters reported, such as 5 or 4 items with low risk of bias, 3 or 2 moderate risks of bias, and 1 or 0 high risks of bias. Graphical analysis was performed using RevMan 5.3 software.

REFERENCES

1. Page, M.J.; Moher, D.; Bossuyt, P.M.; Boutron, I.; Hoffmann, T.C.; Mulrow, C.D.; Shamseer, L.; Tetzlaff, J.M.; Akl, E.A.; Brennan, S.E.; et al. PRISMA 2020 explanation and elaboration: Updated guidance and exemplars for reporting systematic reviews. *BMJ* **2021**, *372*, doi:10.1136/bmj.n160.

2. Sarkis-Onofre, R.; Skupien, J.A.; Cenci, M.S.; Moraes, R.R.; Pereira-Cenci, T. The role of resin cement on bond strength of glass-fiber posts luted into root canals: A systematic review and metaanalysis of in vitro studies. *Oper. Dent.* **2014**, *39*, 31–44, doi:10.2341/13-070-LIT.
