# Supplementary material for: Bacterial Adhesion Strength on Titanium Surfaces Quantified by Atomic Force Microscopy: A Systematic Review
Source: Antibiotics (Basel). 2023 Jun 1;12(6):994. doi: 10.3390/antibiotics12060994 (PMC10295333; doi:10.3390/antibiotics12060994)
Supplement: Supplementary file 1 [file antibiotics-12-00994-s001.zip › Supplemental Table S1.pdf]

Table S1. Databases and search strategy

Database	Search	Found
**EMBASE** June 21st, 2022	(titanium OR 'titanium alloy' OR 'Ti alloy' OR 'dental implant' OR implant) AND ('atomic force microscopy' OR 'AFM' OR 'atomic force spectroscopy' OR 'simply force spectroscopy') AND ('bacterial adhesion' OR 'bacterial attachment')	128
**PubMed** June 21st, 2022	(titanium OR "titanium alloy" OR "Ti alloy" OR "dental implant" OR implant) AND ("atomic force microscopy" OR "AFM" OR "atomic force spectroscopy" OR "simply force spectroscopy") AND ("bacterial adhesion" OR "bacterial attachment")	135
**Scopus** June 21st, 2022	(titanium OR "titanium alloy" OR "Ti alloy" OR "dental implant" OR implant) AND ("atomic force microscopy" OR "AFM" OR "atomic force spectroscopy" OR "simply force spectroscopy") AND ("bacterial adhesion" OR "bacterial attachment")	162
**Science Direct** June 21st, 2022	(titanium OR "titanium alloy" OR implant) AND ("atomic force microscopy" OR "AFM" OR "atomic force spectroscopy" OR "simply force spectroscopy") AND ("bacterial adhesion" OR "bacterial attachment")	838

Grey literature

**ProQuest Dissertations and Theses** June 21st, 2022	(titanium OR "titanium alloy" OR "Ti alloy" OR "dental implant" OR implant) AND ("atomic force microscopy" OR "AFM" OR "atomic force spectroscopy" OR "simply force spectroscopy") AND ("bacterial adhesion" OR "bacterial attachment")	100
**Google Scholar** June 21st, 2022	(titanium OR "titanium alloy" OR "Ti alloy" OR "dental implant" OR implant) AND ("atomic force microscopy" OR "AFM" OR "atomic force spectroscopy" OR "simply force spectroscopy") AND ("bacterial adhesion" OR "bacterial attachment")	100
