# Supplementary material for: Bacterial Adhesion Strength on Titanium Surfaces Quantified by Atomic Force Microscopy: A Systematic Review
Source: Antibiotics (Basel). 2023 Jun 1;12(6):994. doi: 10.3390/antibiotics12060994 (PMC10295333; doi:10.3390/antibiotics12060994)
Supplement: Supplementary file 1 [file antibiotics-12-00994-s001.zip › Supplemental Table S2.pdf]

Table S2. Excluded articles and reasons for exclusion.

Author, year	Reason for exclusion
Ahimou et al., 2007	1
Almaguer-Flores et al., 2010	2
Badea et al., 2016	2
Barbour et al., 2007	3
Bazaka et al., 2020	2
Beltrán-Partida et al., 2015	3
Beltrán-Partida et al., 2016	2
Boshkovikj et al., 2014	2
Braceras et al., 2014	2
Brunetto and Fromm, 2010	4
Caro-Lara et al., 2021	5
Cunha et al., 2016	2
Es-Souni et al., 2021	2
Nancy and Rajendran, 2018	2
del Olmo et al., 2021	2
Menzel et al., 2021	4
Dong et al., 2017	2
Elschner et al., 2016	2
Ercan et al., 2011	2
Farrokhi-Rad et al., 2018	2
Gadenne et al., 2013	3
Gambardella et al., 2021	2
Golda-Cepa et al., 2015	2
Gonzalez et al., 2020	2
Gopal et al., 2016	2
Govindharajulu et al., 2017	2
Badihi Hauslich et al., 2013	6
Hofherr et al., 2020	6
Hou et al., 2022	6
Ivanova et al., 2010	2
Jasevičius et al., 2015	7
Jhong et al., 2020	2
Kunrath et al., 2020	2
Liu et al., 2015	2
Liu et al., 2016	2
Lüdecke et al., 2013	8
Lüdecke et al., 2016	2
Matos et al., 2017	2
Na et al., 2010	2
Nie et al., 2017	2
Muhammad Imran Rahim et al., 2021	4
M I Rahim et al., 2021	9

Roberts et al., 2013	3
Singh et al. 2011	2
Singh et al., 2013	3
Skovager et al., 2013	10
Vadillo-Rodríguez et al., 2018	11
Verran et al., 2010	12
Wassmann et al., 2017	2
Whitehead et al., 2006	12
Whitehead et al., 2005	12
Ye et al., 2022	2

1) Evaluate biofilm adhesion (n=1), 2) AFM analysis surface topography (n=31); 3) AFM analysis biofilm formation on substrate (n=5), 4) Conference Abstract (n=3); 5) Copper sheets (n=1); 6) Do not use AFM to study adhesion forces of bacteria on substrate (n=3); 7) AFM analysis molecular surface characteristics of bacteria (n=1); 8) Glass slides (n=1); 9) Did not correlate adhesion strength to roughness and wettability (n=1); 10) Stainless steel substrate (n=1); 11) Surface patterns (n=1); 12) silicon wafers (n=3).

REFERENCES

Ahimou, F., Semmens, M.J., Novak, P.J., Haugstad, G., 2007. Biofilm cohesiveness measurement using a novel atomic force microscopy methodology. Appl. Environ. Microbiol. 73, 2897–2904. https://doi.org/10.1128/AEM.02388-06

Almaguer-Flores, A., Ximénez-Fyvie, L.A., Rodil, S.E., 2010. Oral bacterial adhesion on amorphous carbon and titanium films: Effect of surface roughness and culture media. J. Biomed. Mater. Res. - Part B Appl. Biomater. 92, 196–204. https://doi.org/10.1002/jbm.b.31506

Badea, M., Braic, M., Kiss, A., Moga, M., Pozna, E., Pana, I., Vladescu, A., 2016. Influence of Ag content on the antibacterial properties of SiC doped hydroxyapatite coatings. Ceram. Int. 42, 1801–1811. https://doi.org/10.1016/j.ceramint.2015.09.143

Badihi Hauslich, L., Sela, M.N., Steinberg, D., Rosen, G., Kohavi, D., 2013. The adhesion of oral bacteria to modified titanium surfaces: Role of plasma proteins and electrostatic forces. Clin. Oral Implants Res. 24, 49–56. https://doi.org/10.1111/j.1600-0501.2011.02364.x

Barbour, M.E., O'Sullivan, D.J., Jenkinson, H.F., Jagger, D.C., 2007. The effects of polishing methods on surface morphology, roughness and bacterial colonisation of titanium abutments. J. Mater. Sci. Mater. Med. 18, 1439–1447. https://doi.org/10.1007/s10856-007-0141-2

Bazaka, O., Bazaka, K., Truong, V.K., Levchenko, I., Jacob, M. V., Estrin, Y., Lapovok, R., Chichkov, B., Fadeeva, E., Kingshott, P., Crawford, R.J., Ivanova, E.P., 2020. Effect of titanium surface topography on plasma deposition of antibacterial polymer coatings. Appl. Surf. Sci. 521, 146375. https://doi.org/10.1016/j.apsusc.2020.146375

Beltrán-Partida, E., Valdez-Salas, B., Escamilla, A., Curiel, M., Valdez-Salas, E., Nedev, N., Bastidas, J.M., 2016. Disinfection of titanium dioxide nanotubes using super-oxidized water decrease bacterial viability without disrupting osteoblast behavior. Mater. Sci. Eng. C 60, 239–245. https://doi.org/10.1016/j.msec.2015.11.042

Beltrán-Partida, E., Valdez-Salas, B., Escamilla, A., Moreno-Ulloa, A., Burtseva, L., Valdez-Salas, E., Curiel Alvarez, M., Nedev, N., 2015. The Promotion of Antibacterial Effects of Ti6Al4V Alloy Modified with TiO2 Nanotubes Using a Superoxidized Solution. J. Nanomater. 2015. https://doi.org/10.1155/2015/818565

Boshkovikj, V., Fluke, C.J., Crawford, R.J., Ivanova, E.P., 2014. Three-dimensional visualization of nanostructured surfaces and bacterial attachment using Autodesk® Maya®. Sci. Rep. 4, 1–6. https://doi.org/10.1038/srep04228

Braceras, I., Vera, C., Ayerdi-Izquierdo, A., Muñoz, R., Lorenzo, J., Alvarez, N., De Maeztu, M.Á., 2014. Ion implantation induced nanotopography on titanium and bone cell adhesion. Appl. Surf. Sci. 310, 24–30. https://doi.org/10.1016/j.apsusc.2014.03.118

Brunetto, P.S., Fromm, K.M., 2010. New antibacterial coated surfaces for self-protecting implants. Eur. Cells Mater. 20, 29.

Caro-Lara, L., Ramos-Moore, E., Vargas, I.T., Walczak, M., Fuentes, C., Gómez, A. V., Barrera, N.P., Castillo, J., Pizarro, G., 2021. Initial adhesion suppression of biofilm-forming and copper-tolerant bacterium Variovorax sp. on laser microtextured copper surfaces. Colloids Surfaces B Biointerfaces 202. https://doi.org/10.1016/j.colsurfb.2021.111656

Cunha, A., Elie, A.M., Plawinski, L., Serro, A.P., Botelho Do Rego, A.M., Almeida, A., Urdaci, M.C., Durrieu, M.C., Vilar, R., 2016. Femtosecond laser surface texturing of titanium as a method to reduce the adhesion of Staphylococcus aureus and biofilm formation. Appl. Surf. Sci. 360, 485–493. https://doi.org/10.1016/j.apsusc.2015.10.102

del Olmo, J.A., Pérez-Álvarez, L., Pacha-Olivenza, M.Á., Ruiz-Rubio, L., Gartziandia, O., Vilas-Vilela, J.L., Alonso, J.M., 2021. Antibacterial catechol-based hyaluronic acid, chitosan and poly (N-vinyl pyrrolidone) coatings onto Ti6Al4V surfaces for application as biomedical implant. Int. J. Biol. Macromol. 183, 1222–1235. https://doi.org/10.1016/j.ijbiomac.2021.05.034

Dong, Y., Ye, H., Liu, Y., Xu, L., Wu, Z., Hu, X., Ma, J., Pathak, J.L., Liu, J., Wu, G., 2017. pH dependent silver nanoparticles releasing titanium implant: A novel therapeutic approach to control peri-implant infection. Colloids Surfaces B Biointerfaces 158, 127–136. https://doi.org/10.1016/j.colsurfb.2017.06.034

Elschner, T., Lüdecke, C., Kalden, D., Roth, M., Löffler, B., Jandt, K.D., Heinze, T., 2016. Zwitterionic Cellulose Carbamate with Regioselective Substitution Pattern: A Coating Material Possessing Antimicrobial Activity. Macromol. Biosci. 16, 522–534. https://doi.org/10.1002/mabi.201500349

Ercan, B., Taylor, E., Alpaslan, E., Webster, T.J., 2011. Diameter of titanium nanotubes influences anti-bacterial efficacy. Nanotechnology 22. https://doi.org/10.1088/0957-4484/22/29/295102

Es-Souni, Mona, Es-Souni, Martha, Bakhti, H., Gülses, A., Fischer-Brandies, H., Açil, Y., Wiltfang, J., Flörke, C., 2021. A bacteria and cell repellent zwitterionic polymer coating on titanium base substrates towards smart implant devices. Polymers (Basel). 13. https://doi.org/10.3390/polym13152472

Farrokhi-Rad, M., Fateh, A., Shahrabi, T., 2018. Electrophoretic deposition of vancomycin loaded halloysite nanotubes-chitosan nanocomposite coatings. Surf. Coatings Technol. 349, 144–156. https://doi.org/10.1016/j.surfcoat.2018.05.070

Gadenne, V., Lebrun, L., Jouenne, T., Thebault, P., 2013. Antiadhesive activity of ulvan polysaccharides covalently immobilized onto titanium surface. Colloids Surfaces B Biointerfaces 112, 229–236. https://doi.org/10.1016/j.colsurfb.2013.07.061

Gambardella, A., Marchiori, G., Maglio, M., Russo, A., Rossi, C., Visani, A., Fini, M., 2021. Determination of the spatial anisotropy of the surface microstructures of different implant materials: An atomic force microscopy study. Materials (Basel). 14. https://doi.org/10.3390/ma14174803

Golda-Cepa, M., Brzychczy-Wloch, M., Engvall, K., Aminlashgari, N., Hakkarainen, M., Kotarba, A., 2015. Microbiological investigations of oxygen plasma treated parylene C surfaces for metal implant coating. Mater. Sci. Eng. C 52, 273–281. https://doi.org/10.1016/j.msec.2015.03.060

Gonzalez, A., Miñán, A.G., Grillo, C.A., Prieto, E.D., Schilardi, P.L., Fernández Lorenzo de Mele, M.A., 2020. Characterization and antimicrobial effect of a bioinspired thymol coating formed on titanium surface by one-step immersion treatment. Dent. Mater. 36, 1495–1507. https://doi.org/10.1016/j.dental.2020.09.006

Gopal, J., Chun, S., Doble, M., 2016. Attenuated total reflection fourier transform infrared spectroscopy towards disclosing mechanism of bacterial adhesion on thermally stabilized titanium nano-interfaces. J. Mater. Sci. Mater. Med. 27, 1–12. https://doi.org/10.1007/s10856-016-5739-9

Govindharajulu, J.P., Chen, X., Li, Y., Rodriguez-Cabello, J.C., Battacharya, M., Aparicio, C., 2017. Chitosan-recombinamer layer-by-layer coatings for multifunctional implants. Int. J. Mol. Sci. 18, 1–16. https://doi.org/10.3390/ijms18020369

Hofherr, L., Müller-Renno, C., Ziegler, C., 2020. FluidFM as a tool to study adhesion forces of bacteria - Optimization of parameters and comparison to conventional bacterial probe Scanning Force Spectroscopy. PLoS One 15, 1–15. https://doi.org/10.1371/journal.pone.0227395

Hou, W., Wu, S., Liu, Y., Li, H., 2022. Impact of conformational change of immunoglobulin G induced by silver ions on Escherichia coli and macrophage adhesion to biomaterial surfaces. Colloids Surfaces A Physicochem. Eng. Asp. 643, 128700. https://doi.org/10.1016/j.colsurfa.2022.128700

Ivanova, E.P., Truong, V.K., Wang, J.Y., Bemdt, C.C., Jones, R.T., Yusuf, I.I., Peake, I., Schmidt, H.W., Fluke, C., Barnes, D., Crawford, R.J., 2010. Impact of nanoscale roughness of titanium thin film surfaces on bacterial Retention. Langmuir 26, 1973–1982. https://doi.org/10.1021/la902623c

Jasevičius, R., Baronas, R., Kačianauskas, R., Šimkus, R., 2015. Numerical modeling of bacterium-surface interaction by applying DEM. Procedia Eng. 102, 1408–1414. https://doi.org/10.1016/j.proeng.2015.01.273

Jhong, Y.T., Chao, C.Y., Hung, W.C., Du, J.K., 2020. Effects of various polishing techniques on the surface characteristics of the ti-6al-4v alloy and on bacterial adhesion. Coatings 10, 1–23. https://doi.org/10.3390/coatings10111057

Kunrath, M., dos Santos, R., de Oliveira, S., Hubler, R., Sesterheim, P., Teixeira, E., 2020. Osteoblastic Cell Behavior and Early Bacterial Adhesion on Macro-, Micro-, and Nanostructured Titanium Surfaces for Biomedical Implant Applications. Int. J. Oral Maxillofac. Implants 35, 773–781. https://doi.org/10.11607/jomi.8069

Liu, W., Su, P., Chen, S., Wang, N., Wang, J., Liu, Y., Ma, Y., Li, H., Zhang, Z., Webster, T.J., 2015. Antibacterial and osteogenic stem cell differentiation properties of photoinduced TiO2 nanoparticle-decorated TiO2 nanotubes. Nanomedicine 10, 713–723. https://doi.org/10.2217/nnm.14.183

Liu, Z., Ma, S., Duan, S., Xuliang, D., Sun, Y., Zhang, Xi, Xu, X., Guan, B., Wang, C., Hu, M., Qi, X., Zhang, Xu, Gao, P., 2016. Modification of Titanium Substrates with Chimeric Peptides Comprising Antimicrobial and Titanium-Binding Motifs Connected by Linkers to Inhibit Biofilm Formation. ACS Appl. Mater. Interfaces 8, 5124–5136. https://doi.org/10.1021/acsami.5b11949

Lüdecke, C., Bossert, J., Roth, M., Jandt, K.D., 2013. Physical vapor deposited titanium thin films for biomedical applications: Reproducibility of nanoscale surface roughness and microbial adhesion properties. Appl. Surf. Sci. 280, 578–589. https://doi.org/10.1016/j.apsusc.2013.05.030

Lüdecke, C., Roth, M., Yu, W., Horn, U., Bossert, J., Jandt, K.D., 2016. Nanorough titanium surfaces reduce adhesion of Escherichia coli and Staphylococcus aureus via nano adhesion points. Colloids Surfaces B Biointerfaces 145, 617–625. https://doi.org/10.1016/j.colsurfb.2016.05.049

Matos, A.O., Ricomini-Filho, A.P., Beline, T., Ogawa, E.S., Costa-Oliveira, B.E., de Almeida, A.B., Nociti Junior, F.H., Rangel, E.C., da Cruz, N.C., Sukotjo, C., Mathew, M.T., Barão, V.A.R., 2017. Three-species biofilm model onto plasma-treated titanium implant surface. Colloids Surfaces B Biointerfaces 152, 354–366. https://doi.org/10.1016/j.colsurfb.2017.01.035

Menzel, H., Chemistry, T., Braunschweig, T.U., 2021. Enzyme-responsive nanoparticles and coatings as drug release systems to fight implant associated infections. Biomed. Eng. / Biomed. Tech. 66, 6–10. https://doi.org/10.1515/bmt-2021-6002

Na, C., McNamara, C.J., Konkol, N.R., Bearce, K.A., Mitchell, R., Martin, S.T., 2010. The use of force-volume microscopy to examine bacterial attachment to titanium surfaces. Ann. Microbiol. 60, 495–502. https://doi.org/10.1007/s13213-010-0078-4

Nancy, D., Rajendran, N., 2018. Vancomycin incorporated chitosan/gelatin coatings coupled with TiO2–SrHAP surface modified cp-titanium for osteomyelitis treatment. Int. J. Biol. Macromol. 110, 197–205. https://doi.org/10.1016/j.ijbiomac.2018.01.004

Nie, B., Long, T., Li, H., Wang, X., Yue, B., 2017. A comparative analysis of antibacterial properties and inflammatory responses for the KR-12 peptide on titanium and PEGylated titanium surfaces. RSC Adv. 7, 34321–34330. https://doi.org/10.1039/c7ra05538b

Rahim, M I, Doll, K., Stiesch, M., Eisenburger, M., 2021. Commensal microflora: A novel therapeutic approach to prevent biomaterial-related infections. Biomed. Tech. 66, S80. https://doi.org/10.1515/bmt-2021-6015

Rahim, Muhammad Imran, Doll, K., Stumpp, N.S., Eisenburger, M., Stiesch, M., 2021. Multilayered Adsorption of Commensal Microflora on Implant Surfaces: an Unconventional and Innovative Method to Prevent Bacterial Infections Associated with Biomaterials. Adv. Mater. Interfaces 8, 1–13. https://doi.org/10.1002/admi.202101410

Roberts, J.L., Khan, S., Emanuel, C., Powell, L.C., Pritchard, M.F., Onsøyen, E., Myrvold, R., Thomas, D.W., Hill, K.E., 2013. An in vitro study of alginate oligomer therapies on oral biofilms. J. Dent. 41, 892–899. https://doi.org/10.1016/j.jdent.2013.07.011

Singh, A.V., Galluzzi, M., Borghi, F., Indrieri, M., Vyas, V., Podestà, A., Gade, W.N., 2013. Interaction of bacterial cells with cluster-assembled nanostructured titania surfaces: An Atomic Force Microscopy study. J. Nanosci. Nanotechnol. https://doi.org/10.1166/jnn.2013.6727

Singh, A.V., Vyas, V., Patil, R., Sharma, V., Scopelliti, P.E., Bongiorno, G., Podestà, A., Lenardi, C., Gade, W.N., Milani, P., 2011. Quantitative characterization of the influence of the nanoscale morphology of nanostructured surfaces on bacterial adhesion and biofilm formation. PLoS One 6. https://doi.org/10.1371/journal.pone.0025029

Skovager, A., Whitehead, K., Wickens, D., Verran, J., Ingmer, H., Arneborg, N., 2013. A comparative study of fine polished stainless steel, TiN and TiN/Ag surfaces: Adhesion and attachment strength of Listeria monocytogenes as well as anti-listerial effect. Colloids Surfaces B Biointerfaces 109, 190–196. https://doi.org/10.1016/j.colsurfb.2013.03.044

Vadillo-Rodríguez, V., Guerra-García-Mora, A.I., Perera-Costa, D., Gónzalez-Martín, M.L., Fernández-Calderón, M.C., 2018. Bacterial response to spatially organized microtopographic surface patterns with nanometer scale roughness. Colloids Surfaces B Biointerfaces 169, 340–347. https://doi.org/10.1016/j.colsurfb.2018.05.038

Verran, J., Packer, A., Kelly, P.J., Whitehead, K.A., 2010. Use of the atomic force microscope to determine the strength of bacterial attachment to grooved surface features. J. Adhes. Sci. Technol. 24, 2271–2285. https://doi.org/10.1163/016942410X508019

Wassmann, T., Kreis, S., Behr, M., Buergers, R., 2017. The influence of surface texture and wettability on initial bacterial adhesion on titanium and zirconium oxide dental implants. Int J Implant Dent 3.

Whitehead, K.A., Colligon, J., Verran, J., 2005. Retention of microbial cells in substratum surface features of micrometer and sub-micrometer dimensions. Colloids Surfaces B Biointerfaces 41, 129–138. https://doi.org/10.1016/j.colsurfb.2004.11.010

Whitehead, K.A., Rogers, D., Colligon, J., Wright, C., Verran, J., 2006. Use of the atomic force microscope to determine the effect of substratum surface topography on the ease of bacterial removal. Colloids Surfaces B Biointerfaces 51, 44–53. https://doi.org/10.1016/j.colsurfb.2006.05.003

Ye, J., Li, B., Zheng, Y., Wu, S., Chen, D., Han, Y., 2022. Eco-friendly bacteria-killing by nanorods through mechano-puncture with top selectivity. Bioact. Mater. 15, 173–184. https://doi.org/10.1016/j.bioactmat.2021.11.028
